
JOURNAL INFORMATION
==============================
NLM Title Abbreviation: ScientificWorldJournal
Journal ID: ScientificWorldJournal
NLM Journal ID: 101131163
Journal ID: TSWJ

The Scientific World Journal

ISSN: 2356-6140
EISSN: 1537-744X
Publisher: Wiley

ARTICLE INFORMATION
==============================
PMCID: PMC6084061
PMID: 30147479
DOI: 10.1100/tsw.2001.155
Article ID: 780978
Article version: 1
Subjects: Short Report

Chromatin Structure and U2AF1-RS1 Gene Expression in Embryonic Stem Cells following RA-Induced Apoptosis

Andollo N. *
Boyano M.D.
Garcia-Sanz M.
Asumendi A.
Zalduendo M.M.
Arechaga J.
Department of Cell Biology, University of the Basque Country, E-48940 Leioa, Spain
*N. Andollo: gcbanvin@lg.ehu.es
Publication date: 2001
Electronic publication date: 2001 Nov 8
Volume: 1
First page: 37
Last page: 37
Copyright: Copyright © 2001 N. Andollo et al.
Copyright year: 2001
License: This is an open access article distributed under the Creative Commons Attribution License, which permits unrestricted use, distribution, and reproduction in any medium, provided the original work is properly cited.
License URL: https://creativecommons.org/licenses/by/3.0/

Funding: University of the Basque Country

==============================
Miami *Nature Biotechnology* Short Reports
*TheScientificWorld* (2001) 1(S3), 37SR
ISSN 1532-2246; DOI 10.1100/tsw.2001.155

### CHROMATIN STRUCTURE AND *U2af1-rs1* GENE EXPRESSION IN EMBRYONIC STEM CELLS FOLLOWING RA–INDUCED APOPTOSIS

N. Andollo*, M.D. Boyano, M. García-Sanz, A. Asumendi, M.M. Zalduendo, and J. Aréchaga

Department of Cell Biology, University of the Basque Country, E-48940 Leioa, Spain
* gcbanvin@lg.ehu.es

**INTRODUCTION.** Apoptosis is a genetically controlled, selective cellular death mechanism, which occurs during processes of differentiation and normal cellular development. AB1 embryonic stem cells were employed in our assays, based on previous studies where it was demonstrated that *in vitro* treatment with retinoic acid (RA) gave rise to apoptosis as well as cellular differentiation (1). In order to support the hypothesis that apoptosis is an active process even when apoptotic bodies appear, we decided to study the chromatin conformation of DNA (2) and the corresponding gene expression. To this end, we analyzed the *U2af1-rs1* gene, which takes part in mRNA splicing.

**METHODS.** AB1 cells after *in vitro* incubation with 1 μM RA for 48 and 72 hours, were stained with propidium iodine and analyzed by flow cytometry. Electron microscopy was employed to verify the ultrastructure of differentiated and apoptotic cells. In order to perform chromatin structure analysis, nuclei were isolated from non-differentiated and differentiated AB1 cells and from apoptotic bodies. After incubation at increasing concentrations of DNase-I, DNA was extracted from the nuclei, digested with *Bgl II*, electrophoresized and hybridized with a mouse *U2af1-rs1* probe. Densitometric measurements on scanned x-ray films were performed with a *Bio-Rad* computer-assisted system. Total RNAs from non-differentiated and differentiated cells and from apoptotic bodies were hybridized with a *U2af1-rs1* probe and with a mouse *Gadph* control probe.

**RESULTS.** Following RA treatment, a decrease of the proliferative population and an increase of the non-proliferative population of AB1 cells was observed. In addition, apoptotical cell death was observed, especially after 72 hours RA-treatment. The ultrastructural phenotype of differentiated AB1 cells after RA-treatment consisted of an increment in the quantity and variety of organelles and the presence of eucromatic nuclei with evident nucleoli. The apoptotical fraction showed a characteristic morphology with cytoplasmic condensation and margination of the hypercondensed chromatin towards the nuclear periphery. Chromatin structure analysis of the *U2af1-rs1* region revealed that differentiated cell DNA was more sensitive to DNase-I than undifferentiated cell DNA. Furthermore, we detected DNase-I sensitivity in apoptotic bodies. Northern blot analysis demonstrated that undifferentiated and differentiated cells and, even apoptotic bodies, expressed the *U2af1-rs1* mRNA.

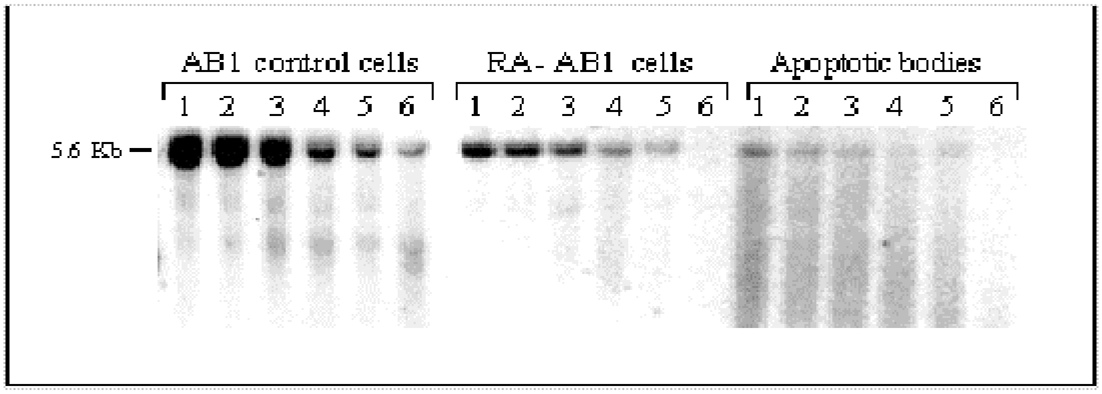

**Fig. 1.** Southern blot showing DNase-I sensitivity in AB1 undifferentiated cells (control cells), RA-differentiated cells and apoptotic bodies. Lanes 1-6 correspond to DNAs extracted from nuclei incubated with increasing concentrations of DNase-I (0, 50, 100, 200, 350 and 500 units/ml) and then, digested with *Bgl II*, to generate DNA fragments of *5.6* Kb.

**DISCUSSION.** A difference in chromatin structure was detected between non-differentiated and differentiated AB1 cells, since differentiated cells were more sensitive to DNase-I digestion in the *U2af1-rs1* region, indicating an "open" chromatin conformation (3). This result, in agreement with the presence of eucromatic nuclei observed by electron microscopy, suggests that differentiated AB1 cells are more active than their non-differentiated counterparts. In contrast to our expectations concerning the apoptotic bodies, we observed that their DNA was DNase-I sensitive. This data indicates that, despite the highly closed conformation of chromatin in apoptotic bodies, both protein accessibility to DNA and *U2af1-rs1* mRNA expression are maintained.

**ACKNOWLEDGEMENT.** This work was supported by grants from the Basque Government and the University of the Basque Country. N. Andollo was supported by a fellowship from the Spanish Education and Culture Ministry.

**REFERENCES.**

1. Atencia, R., Garcia-Sanz, M., Unda, F., and Arechaga, J. (1994) Exp. Cell Res. 214(2), 663-667
2. Khodarev, N.N, Sokolova, I.A., and Vaughan, A.T.M. (1998) Int. J. Radiat. Biol. 73(5), 455-467
3. Feil, R., Boyano, B.D., Allen, N.D., and Kelsey, G. (1997) J. Biol. Chem. 272, 20893-20900
